# Correlation analysis of monocyte subsets and insulin resistance considering fetuin-A involvement in patients with type 2 diabetes

**DOI:** 10.1186/s40169-018-0187-4

**Published:** 2018-03-27

**Authors:** Saori Maruo, Katsuhito Mori, Koka Motoyama, Miyuki Nakamura, Reina Kawarabayashi, Yoshinori Kakutani, Yuko Yamazaki, Tomoaki Morioka, Tetsuo Shoji, Masaaki Inaba, Masanori Emoto

**Affiliations:** 1 Department of Metabolism, Endocrinology and Molecular Medicine, Osaka City University Graduate School of Medicine, Osaka, Japan; 2 Department of Nephrology, Osaka City University Graduate School of Medicine, 1-4-3, Asahi-machi, Abeno-ku, Osaka, 545-8585 Japan; 3 Department of Vascular Medicine, Osaka City University Graduate School of Medicine, Osaka, Japan

**Keywords:** Fetuin-A, Insulin resistance, CD14, CD16, Type 2 diabetes, Monocyte subsets

## Abstract

**Background:**

Fetuin-A is a multifunctional circulating glycoprotein that can induce insulin resistance. Lately, adipose tissue has gained prominence as an effector site of fetuin-A. Although fetuin-A—induced proinflammatory polarization and migration of macrophages plays a crucial role, it remains obscure whether monocyte subsets in circulation could simulate characteristics of macrophages in adipose tissues. This study aims to investigate the correlation between monocyte subsets with fetuin-A and its relevant insulin resistance.

**Results:**

We evaluated serum fetuin-A levels in 107 patients with type 2 diabetes (T2D). Using flow cytometry, we classified monocyte subsets into three subtypes: (a) classical, CD14^++^CD16^−^; (b) intermediate, CD14^++^CD16^+^, the most proinflammatory one; (c) and nonclassical, CD14^+^CD16^++^. We assessed the insulin resistance by the homeostasis model assessment for insulin resistance (HOMA-IR) in 68 patients without insulin injections. We observed no correlation between fetuin-A levels and classical (*ρ* = − 0.005; *P* = 0.959), intermediate (*ρ* = 0.022; *P* = 0.826), and nonclassical monocyte counts (*ρ* = 0.063; *P* = 0.516), respectively. In addition, no significant correlation was found between log (HOMA-IR) and classical (*ρ* = 0.052; *P* = 0.688), intermediate (*ρ* = 0.054; *P* = 0.676), and nonclassical monocyte counts (*ρ* = 0.012; *P* = 0.353), respectively. However, serum fetuin-A levels showed positive correlation with log (HOMA-IR) (*ρ* = 0.340; *P* = 0.007). Multiple regression analyses revealed a significant relationship between fetuin-A and log (HOMA-IR) (*β* = 0.313; *P* = 0.016), but not with monocyte subsets.

**Conclusions:**

Monocyte subsets in circulation, including proinflammatory intermediate monocytes, were not associated with fetuin-A and insulin resistance.

## Background

Fetuin-A (α2-Heremans Schmid glycoprotein: AHSG) is an abundant circulating glycoprotein that is primarily synthesized in the liver and plays several functions in human physiology and pathology [1]. Among these, insulin resistance induction is well recognized [2, 3]. Recently, adipose tissue, which is the primary inflammation site where infiltrated macrophages interact with adipocytes, has gained prominence as an effector site of fetuin-A. Pal et al. demonstrated that fetuin-A acts as an essential adaptor protein when free fatty acids (FFA) bind to Toll-like receptor-4 (TLR4) in both adipocytes and macrophages, signifying that FFA–fetuin-A–TLR4 ternary complex with intracellular activation of NF-κB can provoke systemic insulin resistance by enhancing proinflammatory cytokine secretion [4]. Fetuin-A has been reported to act as a chemoattractant for macrophage migration into adipose tissue and, subsequently, polarize anti-inflammatory M2 to proinflammatory M1 macrophages [5]; this suggests that the pivotal role of fetuin-A in inflamed adipose tissue is a collaboration with macrophages.

Monocytes, originally derived from the bone marrow, circulate in the bloodstream and infiltrate into tissues in response to environmental changes, resulting in differentiated macrophages [6]. Hence, the monocyte–macrophage system is involved in various inflammatory diseases, including atherosclerosis [6]. Although monocytes are heterogeneous and diverse, various markers and different terms have confused monocyte classification. Accordingly, a consensus nomenclature for monocytes was proposed and approved, internationally, in 2010 to solve this problem [7]. At present, the following three distinct subsets of human monocytes are recognized according to the expression of CD14, a lipopolysaccharide co-receptor, and CD16, FCγ-III receptor: (a) classical, CD14^++^CD16^−^; (b) intermediate, CD14^++^CD16^+^; and (c) nonclassical, CD14^+^CD16^++^ [7]. In comparison to others, CD14^++^CD16^+^ monocytes show proinflammatory characteristics [8]. In fact, the elevated numbers of CD14^++^CD16^+^ monocytes have been reported to be an independent predictor of cardiovascular events [9–11], suggesting a profound association between CD14^++^CD16^+^ monocytes and atherosclerosis.

Although recent evidence supports that fetuin-A can elicit and amplify insulin resistance within adipose tissue, estimation of macrophage-driven adipose tissue inflammation without invasive techniques, such as biopsy, is challenging. We hypothesized that fetuin-A—induced polarization of macrophages from M2 to M1 in adipose tissue might be comparable to the proinflammatory shift of monocyte subsets by fetuin-A in the bloodstream. Hence, this study aims to elucidate whether circulating monocyte subsets, especially CD14^++^CD16^+^ monocytes, are associated with serum fetuin-A levels and their related insulin resistance in patients with type 2 diabetes mellitus (T2D).

## Methods

### Subjects

In this study, we enrolled patients with T2D who admitted to our diabetes center at Osaka City University Hospital (Osaka, Japan) between October 2011 and April 2013. The diagnosis of diabetes was according to a history of diabetes or the American Diabetes Association criteria [12]. Notably, we excluded patients with serum creatinine levels > 1.5 mg/dL and other active medical diseases from this study. We calculated the estimated glomerular filtration rate (eGFR) per the guidelines of the Japanese Society of Nephrology [13]. This study was approved by the ethical review board of our institution (registration number 164), and we obtained informed consent from each patient.

### Biochemical analysis

We determined serum fetuin-A using an enzyme-linked immunosorbent assay kit (BioVender Laboratory Medicine, Inc., Czech Republic) as previously described [14, 15].

### Homeostasis model assessment

Based on Matthews et al. we calculated the Homeostasis model assessment for insulin resistance (HOMA-IR) from fasting plasma glucose (FPG) and immunoreactive insulin (FIRI), using the following formula [16]: HOMA-IR = FIRI in μU/mL × FPG in mmol/L/22.5.

### Peripheral blood monocyte cell isolation and flow cytometry analysis

**Fig. 1:**
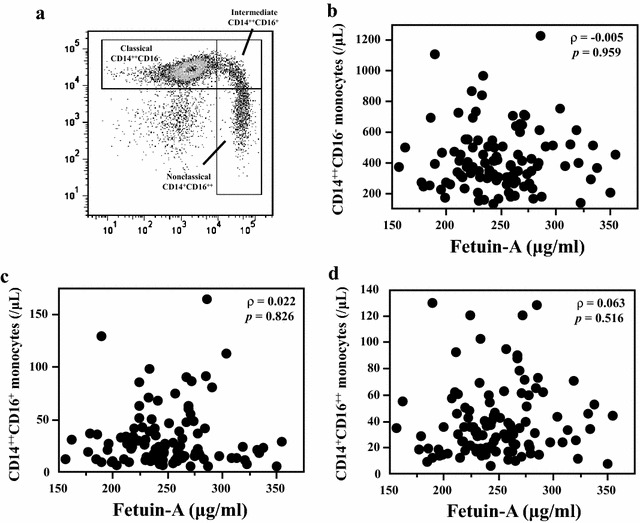
The flow cytometric analysis of monocyte subsets (representative example; **a**) and the association of serum levels of fetuin-A with monocyte subsets (**b**–**d**)

Peripheral blood samples were freshly collected from all patients after 8-h fasting period with ethylenediaminetetraacetic acid—containing (EDTA) tube. Erythrocytes were lysed using a lysing solution (lysing buffer; BD Pharm Lyse^™^; BD Biosciences, Japan) to segregate leukocytes. For the cytometric analysis, we used fluorescent-conjugated monoclonal antibodies against CD14 (PE-Cy7 Mouse Anti-Human CD14; BD Pharmingen^™^, BD Biosciences) and CD16 (APC-Cy7 Mouse Anti-Human CD16; BD Pharmingen^™^, BD Biosciences) for labeling monocyte subsets. We measured 100,000 labeled leukocytes for each patient by flow cytometry (BD FACSCanto^™^; Becton–Dickinson Bioscience, Japan). Monocytes were first gated by the forward scatter and sideward scatter dot plot. After that, according to the properties and the level CD14 and CD16 expression, we categorized monocytes into the following three subsets: (a) classical, CD14^++^CD16^−^; (b) intermediate, CD14^++^CD16^+^; and (c) nonclassical, CD14^+^CD16^++^. We analyzed using FlowJo 7.5.5 (TreeStar Inc.) (Fig. 1a).

### Statistical analysis

In this study, all values are presented as mean ± SD or median (interquartile) as appropriate. Data were evaluated using JMP v.10 (SAS Institute Inc., Cary, NC). In addition, unpaired *t* test and the Mann–Whitney *U*-test were used where appropriate. We used the Spearman’s rank correlation test to evaluate the relationships among serum fetuin-A, monocyte subsets, and HOMA-IR. Log-transformation was performed to achieve a normal distribution of HOMA-IR, and multivariate regression analyses were performed to evaluate the correlation between log (HOMA-IR) and various parameters. We considered *P* < 0.05 as statistically significant.

## Results

Table 1 summarizes the clinical characteristics of the study population. We evaluated serum fetuin-A levels in 107 patients with type 2 diabetes (T2D). The mean serum levels of fetuin-A were 249 ± 40 μg/mL. The median monocyte count was 486 (368–646) cells/μL, of which 379 (283–510) cells/μL were classical CD14^++^CD16^−^ monocytes, 24 (15–39) cells/μL were intermediate CD14^++^CD16^+^ monocytes, and 33 (20–51) cells/μL were nonclassical CD14^+^CD16^++^ monocytes. Of all patients, 39 were had undergone treatment with insulin injections. Regarding fetuin-A levels and monocyte subset counts, no difference was observed with or without insulin therapy.

**Table 1:** Clinical characteristics of the study population

	Without insulin	With insulin	Total
*n*	68	39	107
Age (years)	57 ± 14	68 ± 8*	61 ± 13
Duration (years)	7 (0–50)	20 (0–46)^#^	10 (0–50)
BMI (kg/m^2^)	27 ± 6	25 ± 4*	26 ± 5
SBP (mmHG)	125 ± 18	136 ± 21*	129 ± 20
Cr (mg/dL)	0.8 ± 0.3	0.8 ± 0.2	0.8 ± 0.2
eGFR (mL/min/1.73 m^2^)	79 ± 27	65 ± 18*	74 ± 25
FPG (mg/dL)	119 (67–203)	103 (72–309)	113 (67–309)
HbA1C (%)	8.1 ± 1.8	8.7 ± 1.4	8.3 ± 1.7
LDL-C (mg/dL)	107 ± 38	88 ± 25*	100 ± 35
TG (mg/dL)	111 (50–1919)	101 (46–303)	109 (46–1919)
HDL-C (mg/dL)	40 ± 11	44 ± 12	41 ± 11
HOMA-IR	2.2 (0–8.3)	N/A	N/A
Fetuin-A (μg/mL)	249 ± 37	249 ± 44	249 ± 40
WBC (cells/μL)	5800 (4825–6775)	5400 (4500–7000)	5700 (4800–6800)
Monocyte (cells/μL)	472 (372–648)	492 (350–616)	486 (368–646)
Classical CD14^++^CD16^−^ monocytes (cells/μL)	363 (291–515)	385 (274–508)	379 (283–510)
Intermediate CD14^++^CD16^+^ monocytes (cells/μL)	25 (15–37)	22 (14–48)	24 (15–39)
Nonclassical CD14^+^CD16^++^ monocytes (cells/μL)	32 (20–54)	35 (19–46)	33 (20–51)
OADs: monotherapy			
Biguanide (Met)	9	2	11
Thiazolidine (Pio)	0	0	0
Other OADs	14	4	18
OADS: combination therapy			
Met + other OADs	14	5	19
Pio + other OADs	2	0	2
Met + Pio ± other OADs	5	0	5

Data are presented as mean ± standard deviation, number, or median (interquartile range)

*SBP* systolic blood pressure, *Cr* creatinine, *LDL-C* low-density lipoprotein-cholesterol, *TG* triglyceride, *HDL-C* high-density lipoprotein-cholesterol, *WBC* white blood cells, *OADs* Oral antidiabetic drugs, *Met* metformin, *Pio* pioglitazone

*, ^#^*P* < 0.05 versus group without insulin

**Fig. 2:**
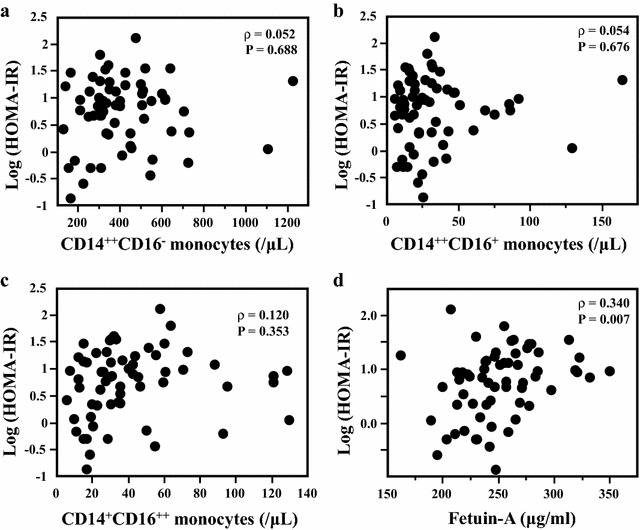
The association of log (HOMA-IR) with monocyte subsets (**a**–**c**) and serum levels of fetuin-A (**d**)

Figure 1b–d shows the correlation between fetuin-A and monocyte subsets. We determined no association between fetuin-A levels and classical CD14^++^CD16^−^ monocyte counts (*ρ* = − 0.005; *P* = 0.959), intermediate CD14^++^CD16^+^ monocyte counts (*ρ* = 0.022; *P* = 0.826), and nonclassical CD14^+^CD16^++^ monocyte counts (*ρ* = 0.063; *P* = 0.516), respectively. Although we divided the subjects into two subgroups (low fetuin-A or high fetuin-A) and performed similar analyses, no significant association was found between fetuin-A and monocyte subsets in each subgroup. Since some medications might affect inflammatory state, serum fetuin-A levels, and distribution of monocyte subsets, we focused on insulin sensitizers (pioglitazone and metformin). However, there was no correlation between fetuin-A and monocyte subsets in patients without insulin sensitizers. After that, we evaluated the association between fetuin-A and monocyte subsets with insulin resistance characterized by HOMA-IR in patients without insulin injection. We determined no significant association between log (HOMA-IR) and classical CD14^++^CD16^−^ monocyte counts (*ρ* = 0.052; *P* = 0.688), intermediate CD14^++^CD16^+^ monocyte counts (*ρ* = 0.054; *P* = 0.676), and nonclassical CD14^+^CD16^++^ monocyte counts (*ρ* = 0.012; *P* = 0.353), respectively (Fig. 2a–c). In addition, no association was determined between log (HOMA-IR) and monocyte subsets in patients without insulin sensitizers. However, serum fetuin-A levels showed positive correlation with log (HOMA-IR) (*ρ* = 0.340; *P* = 0.007; Fig. 2d). We performed multiple regression analyses to explore the impact of various clinical factors on insulin resistance. Although body mass index (BMI; *β* = 0.305; *P* = 0.034) and fetuin-A were significantly associated with log (HOMA-IR) (*β* = 0.313; *P* = 0.016), monocyte subsets were not (Table 2).

**Table 2:** Multivariate regression analyses of clinical factors possibly affecting the insulin resistance (HOMA-IR)

	*β*	*P*
Age	− 0.005	0.975
Sex (Male)	0.150	0.275
BMI	0.305	0.034
CD14^++^CD16^−^ monocytes (/μL)	0.015	0.939
CD14^++^CD16^+^ monocytes (/μL)	0.037	0.871
CD14^+^CD16^++^ monocytes (/μL)	0.145	0.428
Fetuin-A (per 1-SD increase)	0.313	0.016
*R* ^2^	0.219	0.052

*SD* standard deviation

## Discussion

To the best of our knowledge, this is the first study to investigate the correlation of monocyte subsets with fetuin-A and insulin resistance in patients with T2D. This study determined no association of monocyte subsets, including CD14^++^CD16^+^ monocytes, with serum fetuin-A levels and HOMA-IR. Conversely, fetuin-A was found to be significantly contributing to insulin resistance.

Apparently, classical CD14^++^CD16^−^ monocytes, which have the high antimicrobial capacity with phagocytosis of pathogens and production of antimicrobial proteins, leave the bone marrow and, subsequently, invade inflamed tissues and differentiated macrophages and/or dendritic cells. Alternatively, they differentiate into intermediate CD14^++^CD16^+^ monocytes in the circulation. Perhaps, nonclassical CD14^+^CD16^++^ monocytes are differentiated from intermediate monocytes, which monitor the endothelial–blood interface and display a crawling behavior. Assumedly, both intermediate and nonclassical monocytes can invade the endothelium, renewing the macrophages and dendritic cell pool in each tissue, including arterial wall [6]. Of these, intermediate monocytes are characterized as the most proinflammatory because of the active production of the reactive oxygen species and inflammatory cytokines such as tumor necrosis factor (TNF)-α and interleukin (IL)-1β. Furthermore, they demonstrate upregulated chemokine receptors relevant to atherosclerosis and high capacity of oxidized low-density lipoprotein-cholesterol (LDL-C) uptake.

In comparison to the apparent association between intermediate CD14^++^CD16^+^ monocytes and cardiovascular events due to atherosclerosis [9–11], its relationship to metabolic disorders seems weak. Some studies have highlighted the positive correlation between CD16^+^ monocytes and obesity, a representative inflammatory state. Rogacev et al. demonstrated a significant correlation between higher CD16^+^ monocyte counts and higher BMI in healthy subjects [17]. Furthermore, a similar relationship between CD16^+^ monocytes and BMI was observed in obese and obese diabetic patients [18]. Remarkably, weight loss induced by diet intervention or gastric surgery led to a significant reduction in CD16^+^ monocytes [18]. Intriguingly, nonclassical CD14^+^CD16^++^ monocytes correlated with BMI rather than intermediate CD14^++^CD16^+^ monocytes.

Although we anticipated a positive correlation between proinflammatory intermediate monocytes and fetuin-A and insulin resistance, no association was determined in this study. Perhaps, the crucial issue might be whether proinflammatory intermediate monocytes in circulation could correspond to M1 macrophages in inflamed tissues [8]. To date, no apparent data illustrate one-to-one correspondence of monocyte subsets to macrophage polarity, M1 or M2. Recently, Jialal et al. reported the lack of association between plasma fetuin-A and the circulating monocyte TLR4 activity in humans [19]. Hence, it could be proposed that the inflammatory shift of monocyte subsets in circulation might not always be parallel to the proinflammatory polarization of M1 to M2 macrophages in adipose tissue [20]. The reason behind why intermediate monocytes cannot link to adipose tissues in inflamed condition is unclear. Perhaps, one primitive explanation for this condition could be the distance from the arterial wall. Since intermediate monocytes can directly penetrate the arterial wall, they can easily participate in plaque formation through their differentiation into macrophages [6]. Conversely, circulating monocytes might be modulated by various factors, including cytokines, in the lead up to reach adipose tissue, resulting in the discrepancy of proinflammatory characteristics between monocytes and macrophages.

Despite lacking any association between monocyte subsets and insulin resistance, fetuin-A is a negative contributor to the insulin sensitivity. Except for adipose tissue, fetuin-A has the potential to target other insulin-sensitive tissues and can directly inhibit the tyrosine kinase activity of the insulin receptor in the liver and skeletal muscle [21, 22]. Perhaps, this could illustrate the positive correlation between fetuin-A and insulin resistance independently of monocyte subsets and BMI.

There are several limitations to this study. First, the number of subjects was small, although we classified monocyte subsets using flow cytometry. Second, this was a cross-sectional study. Moreover, we mainly targeted obese patients with type 2 diabetes. The lack of lean subjects without diabetes disturbed further analyses. It might hide true relationship between monocyte subsets and inflammatory state. In addition, there was no information about characteristics of macrophages in adipose tissue. Therefore, additional studies are needed to compare circulating monocyte subsets with infiltrated macrophages in adipose tissue evaluated by biopsy.

## Conclusions

To conclude, this study determined no correlation between monocyte subsets, including proinflammatory intermediate monocytes, and fetuin-A. Neither of the monocyte subsets demonstrated correlation with insulin resistance; however, fetuin-A showed a negative association with insulin sensitivity. Overall, this study suggests that monocyte subset counts in circulation could not parallel adipose tissue inflammation.

## Abbreviations

T2D: type 2 diabetes
AHSG: α2-Heremans Schmid glycoprotein
HOMA-IR: homeostasis model assessment for insulin resistance
FFA: free fatty acids
TLR4: Toll-like receptor-4
FPG: fasting plasma glucose
FIRI: fasting immunoreactive insulin
BMI: body mass index
SBP: systolic blood pressure
Cr: creatinine
LDL-C: low-density lipoprotein-cholesterol
TG: triglyceride
HDL-C: high-density lipoprotein-cholesterol
WBC: white blood cells
TNF: tumor necrosis factor
IL: interleukin
